# Supplementary figures and images for: Extinction risk of narrowly distributed species of seed plants in Brazil due to habitat loss and climate change
Source: PeerJ. 2019 Jul 22;7:e7333. doi: 10.7717/peerj.7333 (PMC6657682; doi:10.7717/peerj.7333)

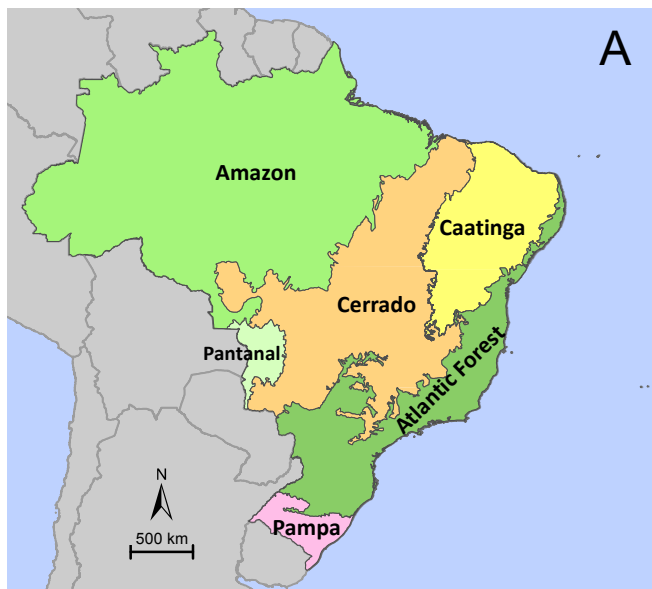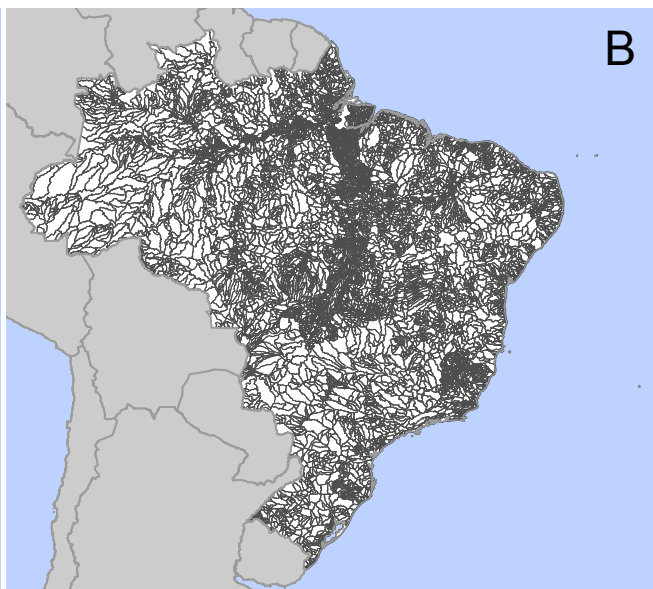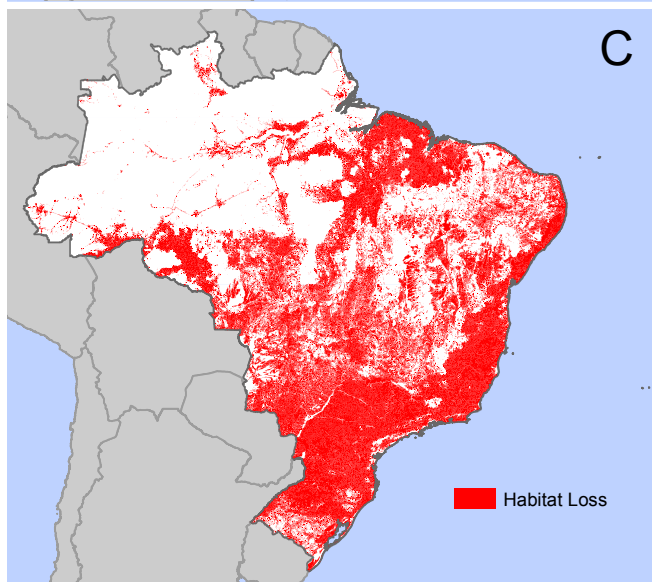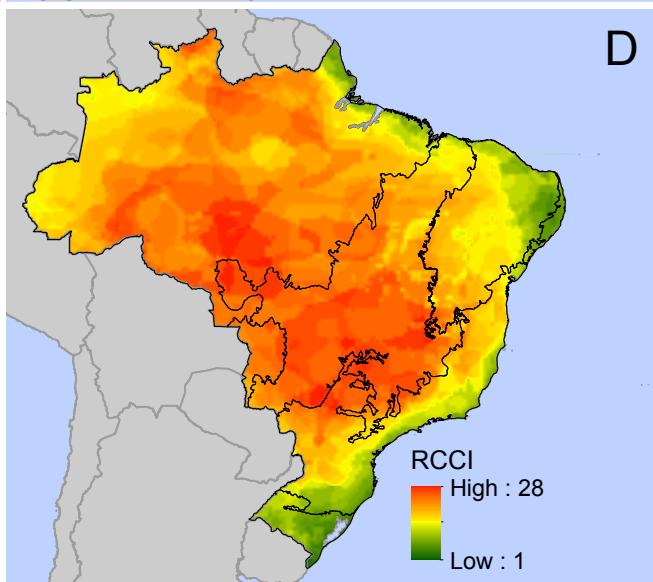

Supplement: Supplemental Information 1 — (a) phytogeographic domains (IBGE, 2004 modified by Silva, Leal & Tabarelli, 2017); (b) fifth-order watersheds (www.metadados.ana.gov.br); (c) cumulative habitat loss in 2014 (IBGE, 2017); (d) Regional Climate Change Index (RCCI). [file peerj-07-7333-s001.pdf]
